# The prevalence of self-reported anxiety, depression, and associated factors among Hanoi Medical University’s students during the first wave of COVID-19 pandemic

**DOI:** 10.1371/journal.pone.0269740

**Published:** 2022-08-12

**Authors:** Dat Tien Nguyen, Tri Minh Ngo, Huong Lan Thi Nguyen, Minh Dai Le, Mai Le Ngoc Duong, Phan Huy Hoang, Ha Viet Nguyen, Kirsty Foster, Tuyen Van Duong, Giang Bao Kim, Tung Thanh Pham

**Affiliations:** 1 Doctor of Medicine program, Hanoi Medical University, Hanoi, Vietnam; 2 School of Preventive Medicine and Public Health, Hanoi Medical University, Hanoi, Vietnam; 3 Academy for Medical Education, The University of Queensland Medical School, Brisbane, Australia; 4 School of Nutrition and Health Sciences, Taipei Medical University, Taipei, Taiwan; 5 Department of Physiology, Hanoi Medical University, Hanoi, Vietnam; International School, Vietnam National University, VIET NAM

## Abstract

**Background:**

Medical students are known to have higher levels of these issues than the general population but in Vietnam the effects of the pandemic on medical student mental health was not documented.

**Objectives:**

To estimate the prevalence and identify factors associated with self-reported anxiety disorder, depression, and perception of worsening mental health among Vietnamese medical students during the COVID-19 pandemic.

**Method:**

A cross-sectional study was conducted from April 7^th^ to 29^th^, 2020. All students in Doctor of General Medicine, Doctor of Preventive Medicine, and Bachelor of Nursing tracks at Hanoi Medical University (3672 students) were invited to participate. Data were collected using an online questionnaire including demographic characteristics, Generalized Anxiety Disorder 7 items, Patient Health Questionnaire 9 items, Fear of COVID-19 scale, and question about worsening mental health status. Robust Poisson regression was used to assess the association between mental health status and associated factors.

**Results:**

Among 1583 students (43.1% response rate), the prevalence of students screened positive for anxiety disorder was 7.3%(95%C.I.:6.0–8.7), depression was 14.5%(95%C.I.:12.8–16.3), and perceiving worsening mental health was 6.9%(95%C.I.:5.7–8.3). In multivariable regression models, significant factors associated with self-reported anxiety disorder included being male (PR = 1.99,95%C.I.:1.35–2.92), difficulty in paying for healthcare services (PR = 2.05,95%C.I.:1.39–3.01), and high level of fear of COVID-19 (Q3:PR = 2.36,95%C.I.:1.38–4.02 and Q4:PR = 4.75,95%C.I.:2.65–8.49). Significant factors associated with self-reported depression were difficulty in paying for healthcare services (PR = 1.78,95%C.I.:1.37–2.30), and high level of fear of COVID-19 (Q3:PR = 1.41,95%C.I.:1.02–1.95 and Q4:PR = 2.23,95%C.I.:1.51–3.29). Significant factors associated with perceived worsening mental health status included having clinical experience (PR = 1.83,95%C.I.:1.17–2.88) and having atypical symptoms of COVID-19 (PR = 1.96,95%C.I.:1.31–2.94).

**Conclusion:**

The prevalence of self-reported depression, anxiety disorder, and worsening mental health among Vietnamese students during the first wave of COVID-19 was lower than in medical students in other countries. Further investigation is needed to confirm this finding.

## Introduction

In late 2019, a novel coronavirus was identified as the cause of a pneumonia case series in Wuhan city, China before it spread all over the world causing a pandemic [1,2]. This “severe acute respiratory syndrome coronavirus 2” (SARS-CoV-2) was shown to be related to the coronavirus of the SARS outbreak in 2003, and the World Health Organization (WHO) declared “COVID-19” as the name of the disease caused by this virus [3]. Until October 4^th^, 2021, the global number of COVID-19 cases was 236 599 025 with 4 831 486 deaths reported across all six WHO regions, while Viet Nam reported 826 837 cases with 20 223 deaths [4].

The rapid spread and severity of COVID-19 has greatly affected public health and significantly stressed the health system, especially the mental health of healthcare workers [5,6]. Research shows that healthcare workers, who work directly with COVID-19 patients or risked exposure to the virus, could experience anxiety about their health status due to lack of personal protective equipment [6]. A study conducted in China showed that health-care workers face significant stress, anxiety, depression, and insomnia during COVID-19 pandemic [5].

Medical students, learning to become future healthcare professionals, are also negatively impacted by the COVID-19 pandemic [6–9]. The pre-pandemic prevalence of depression, depressive symptoms among medical students in 43 countries around the world was 27,2% with 11,1% reporting suicidal ideation [9]. During the pandemic, the mental health status of medical students changed dramatically in some countries. In Nigeria, the proportion of students with severe anxiety is 24% while in Bangladesh, the proportion of students with depressive symptoms is 82.4% and anxiety symptoms 87.7% [7,8].

Previous studies showed that the pandemic negatively affected Vietnamese healthcare workers’ mental health and quality of life [10,11]. Vietnamese healthcare workers were sent to provinces with COVID-19 cases to collect test sample, to quarantined areas to care for patients, or were quarantined if they had been in close contact with confirmed cases without adequate protective equipment [10,11]. Moreover, medical students were also recruited and trained for COVID-19 responses in Vietnam [12]. These students supported the Center for Disease Control in Hanoi and other provinces with epidemiological investigation of close contacts of COVID-19 patients through telephone calls and other available data [12]. In July 2021, when the 4^th^ wave of COVID-19 hit the South of Vietnam, Hanoi Medical University’s students were also mobilized to support Binh Duong province to collect clinical specimens and other COVID-19-related activities[13,14].

Before the pandemic, the prevalence of depression among medical students in Vietnam was 15.2%, which is considerably higher than the 2.8% prevalence of the general population [15]. Despite their participation in pandemic responses, the impact of COVID-19 on the mental health of Vietnamese medical students was not well documented. Therefore, we conducted this study to estimate the prevalence of self-reported depression, anxiety disorder, and perception of worsening mental health as well as identify its associated factors among Hanoi Medical University’s students in three majors: Doctor of General Medicine, Doctor of Preventive Medicine, and Bachelor of Nursing as these majors have primarily been involved in COVID-19 responses in Vietnam.

## Materials and methods

### Study designs and settings

We conducted a cross-sectional study at Hanoi Medical University from April 7^th^ to 29^th^, 2020. Hanoi Medical University is one of the largest medical universities in Vietnam providing both graduate and postgraduate education [15,16]. The university undergraduate training is divided into two groups: 4-year curriculum for health science track (Bachelor of Public Health, Nursing, Medical technology, Nutrition, and Optometry) and 6-year curriculum for medical track (Doctor of General Medicine, Traditional Medicine, Preventive Medicine, and Dental Medicine). Regarding the clinical training at HMU, students begin clinical rotation in hospitals (including overnight duty) in the third-year for the Doctor of General Medicine and Doctor of Preventive Medicine tracks and in the second-year for the Bachelor of Nursing track [15,16]. Further information regarding Vietnam medical education system could be found elsewhere [16].

### Sample and data collection

From April 7^th^ to 29^th^, 2020, we invited all students form the Doctor of General Medicine, Doctor of Preventive Medicine, and Bachelor of Nursing at Hanoi Medical University to participate in our study (3672 students). These majors have primarily been involved in COVID-19 responses in Vietnam. 43.1% of them (1583 students) agreed to participate and completed the research questionnaire, of which 1032 subjects belong to the Doctor of General Medicine program (out of 2921 invited, 35.3%), 308 subjects belong to the Doctor of Preventive Medicine program (out of 466 invited, 66.1%), and 243 subjects belong to the Bachelor of Nursing program (out of 285 invited, 85.3%).

The data was collected using an online anonymous Google Form questionnaire, which included demographic information, medical history, and mental health screening questionnaires. The research team together with Office for Student Services sent the Google Form link on an official university notice to a representative in each class. The representative then delivered the documents to their class’s Facebook group and other social media platform, so students in invited classes could read information regarding the study and decide whether to participate. The research team checked the record and sent reminders to relevant classes weekly. All questions were marked as required, so we had no missing data from submitted questionnaires. At the end of April 2020, the Google Form link was closed, and data was extracted to an Excel file and then converted into Stata data file.

### General questionnaire

The online questionnaire was divided in to four sections: demographic information, academic information, general health screening, and mental health screening. The demographic section collected information about gender (male/female), marital status (single/other (other containing married, divorced, and widow)), and having difficulty in paying for healthcare services (no/yes). Specifically, the question about the affordability to pay for healthcare services had four answers: “very difficult”, “difficult”, “easy”, “very easy”. Students with “very difficult” and “difficult” answers were defined as having difficulty in paying for healthcare services, and the rest (“very easy” and “easy”) were defined as not having difficulty. Academic section included academic majors (Doctor of General Medicine/Doctor of Preventive/Bachelor of Nursing) and having clinical experience (no/yes). Students with clinical experience were classified according to whether they had any clinical rotation experience at the time of the survey.

General health section included Body Mass Index (BMI) classification (underweight/normal/overweight/obese), COVID-19 symptoms (have no symptoms/have only atypical symptoms/have typical symptoms) and having chronic diseases (no/yes). BMI was calculated based on self-reported weight and height through the equation: BMI = weight (kilograms)/height^2^ (meters) [17]. According to WHO classification in Asian population, students with BMI score less than 18.5 were classified as underweight, from 18.5 to less than 23 were classified as normal, from 23 to less than 27.5 were classified as overweight, and the rest (BMI > 27.5) were defined as obese[17]. Regarding COVID-19 symptoms, participants would be classified as having typical symptoms of COVID-19 if they have at least one of the three typical symptoms: fever, cough and shortness of breath [18]. Participants having only atypical symptoms of COVID-19 included those having none of the three typical symptoms and at least one of the atypical symptoms: muscle ache, fatigue, sputum, anxiety, headache, sore throat, runny nose, chest pain, hemorrhage, diarrhea, and vomiting. Finally, if they did not have any of the above symptoms, they would be classified as having no symptoms [18]. This classification of COVID-19 symptoms was based on the Vietnamese health declaration for foreigners and Vietnam citizens at the time of the survey [19]. Regarding chronic diseases, students were categorized as having chronic diseases if they reported previous diagnosis of coronary thrombosis, heart failure, peripheral vascular, cerebrovascular accident, asthma/chronic obstructive pulmonary disease, uncomplicated diabetes, depression, anticoagulant drugs, Alzheimer/dementia hemiplegia, kidney disease, cancer, chronic liver diseases, metastatic cancer, acquired immunodeficiency diseases [20].

### Outcome measures

In the mental health screening section, we collected information on the fear of COVID-19, anxiety disorder screening, depression screening, and perceived mental health status. The Fear of COVID-19 Scale (FCoV-19S) had 7 items developed by Daniel Kwasi Ahorsu and colleagues and was used to describe the level of fear of COVID-19 among general population [21]. This 7-item scale was translated into Vietnamese by the research team and was reviewed by an expert panel (including 1 psychiatrist, 10 medical doctors, 7 nurses, and 5 public health professionals) with the suggestion of keeping the original rating scale and scoring [22]. Each item of the scale had five answers based on Likert-5 scale: 1 = “totally disagree”; 2 = “disagree”; 3 = “Neutral”; 4 = “agree”; 5 = “totally agree”. The total score ranged from 7 to 35 points, and the larger the score the higher the level of fear. In this study, the FCoV-19S score was divided into four groups based on quartiles: Q1, Q2, Q3, and Q4. Q1 group contained 25% participants with the lowest score from 7 to 13 points; Q2 group consisted of 25% participants with medium low score from 14 to 20 points; Q3 group was 25% participants with medium high score from 21 to 27 points; Q4 group was 25% participants with highest score from 28 to 35 points.

Anxiety disorder was screened for using the Generalized Anxiety Disorder– 7 items (GAD-7) questionnaire developed by Robert L. Spitzer and colleagues [23]. GAD-7 is widely used in Vietnam and all over the world [23,24]. For each item, participants had 4 options: 0 = “Never”; 1 = “Several days”; 2 = “More than half of the day”; 3 = “Nearly every day”. The total score ranged from 0 to 21 and was divided into four levels: None (0–4 points), Mild (5–9 points), Moderate (10–14 points), Severe (15–21). In our study, we used 10-point as the cutoff associated with positive screening for anxiety disorder. This 10-point cutoff has 89% sensitivity and 82% specificity [23].

The Patient Health Questionnaire 9 items developed by Robert L. Spitzer and colleagues is used to screen for depression in Vietnam and other countries [25–27]. Each item had four option values: 0 = “Never”; 1 = “Several days”; 2 = “More than half of the day”; 3 = “Nearly every day”. The total score ranged from 0 to 27 points and was divided into five groups: None (0–4 points), Mild (5–9 points), Moderate (10–14 points), Moderately Severe (15–19 points), Severe (20–27 points). Students with 10 points or more were considered as having self-reported depression. This screening cut-off was shown to have 88% sensitivity and 88% specificity [25].

The perception of mental health status (worse/not worse) was assessed by a direct question: “How has your mental health changed from the start of COVID-19” with three answers: “worse”; “unchanged”; “better”. In particular, worsening was defined as feeling that the pandemic negatively affects their mental health while not worsening included “unchanged” and “better” answers.

### Data analysis

The Excel file extracted from Google Form was converted into.dta file (Stata data file), and the data was cleaned and analyzed by Stata 15.1 software. We calculated the frequency and proportion to describe all categorical variables and utilized Chi-squared test to test for differences among these variables. P-value < 0.05 was considered statistically significant in this study.

In this research, due to the high prevalence of self-reported depression and anxiety disorder, logistic regression would potentially overestimate the association between depression or anxiety with other factors [28,29]. In addition, applying log-binominal to directly estimate Prevalence Ratios (PRs) could solve the issue of overestimation by odds ratio, but this type of model usually runs into problems of non-convergence [30]. However, Zou and Barros and colleagues showed that Poisson regression model with robust variance was able to estimate PRs for binary variables [28,29]. Additionally, the result from Poisson regression was approximate to the result from log-binominal model [31]. Therefore, we decided to use Poisson regression with our data.

### Ethical issue

This study was approved by the Biomedical Research Ethics Council of Hanoi Universities of Public Health and the administrative board of Hanoi Medical University (IRB No. 133/2020/YTCC-HD3). All participants received comprehensive information about the survey and so were fully informed before giving consent (by clicking “I agree to participate” button on the informed consent page of the questionnaire). The IRB approved this consent procedure for our online survey. The anonymous questionnaire did not collect identifiable information from the participants, and the participants could refuse to participate and stop filling in the Google Form at any time during the survey.

## Results

Overall, 1583 students (out of 3672 invited students) in Doctor of General Medicine (1032 out of 2921 invited, 35.3%), Doctor of Preventive Medicine (308 out of 466 invited, 66.1%) and Bachelor of Nursing (243 out of 285 invited, 85.3%) agreed to participate in the survey, and the overall response rate was 43.1% among all students (**Table 1)**. The distribution of male and female students was different in academic majors (p < 0.001). In particular, the ratio between male and female participants was approximately 1:1 among General Medicine group and 1:3 among Preventive Medicine group. However, female students accounted for 96.7% of Nursing major (nearly 3:97 ratio). BMI also showed the different distribution among the three groups of academic majors (p < 0.001). Specifically, the General Medicine groups had the highest percentage of overweight and obese (15.8% and 1.9%, respectively) while this proportion among Preventive Medicine group was 12.3% and 1.3% and among Nursing group was 3.3% and 0.8%. On the other hand, General Medicine groups had the lowest percentage of underweight compared to the other groups (16.3% vs. 24.4% vs. 32.5%). Among all participants, 1035 students (65.4%) had clinical experience, among which 61.6% was students in General Medicine, 24.3% was Preventive Medicine, and 14.1% was Nursing (p < 0.001).

**Table 1:** Characteristic of participants.

	General Medicine	Preventive Medicine	Nursing	Total	P-value
n (%)	1032 (65.2)	308 (19.5)	243 (15.4)	1583 (100.0)	
**Demographic factors**					
**Gender, n(%)**					**<0.001** ^**a**^
Female	525 (50.9)	231 (75.0)	235 (96.7)	991 (62.6)	
Male	507 (49.1)	77 (25.0)	8 (3.3)	592 (37.4)	
**Marital status, n(%)**					0.433^b^
Single	1025 (99.3)	304 (98.7)	242 (99.6)	1571 (99.2)	
Other	7 (0.7)	4 (1.3)	1 (0.4)	12 (0.8)	
**Having difficulty in paying for healthcare service, n(%)**					**<0.001** ^**a**^
No	543 (52.6)	179 (58.1)	101 (41.6)	823 (52.0)	
Yes	489 (47.4)	129 (41.9)	142 (58.4)	760 (48.0)	
**Academic factors**					
**Having clinical experience, n(%)**					**<0.001** ^**a**^
No	394 (38.2)	57 (18.5)	97 (39.9)	548 (34.6)	
Yes	638 (61.8)	251 (81.5)	146 (60.1)	1035 (65.4)	
**Health factors**					
**BMI classification, n(%)**					**<0.001** ^**a**^
Underweight	168 (16.3)	75 (24.4)	79 (32.5)	322 (20.3)	
Normal	681 (66.0)	191 (62.0)	154 (63.4)	1026 (64.8)	
Overweight	163 (15.8)	38 (12.3)	8 (3.3)	209 (13.2)	
Obese	20 (1.9)	4 (1.3)	2 (0.8)	26 (1.6)	
**COVID-19 symptoms, n(%)**					**<0.001** ^**a**^
Has no symptoms	864 (83.7)	242 (78.6)	171 (70.4)	1277 (80.7)	
Has only atypical symptoms	137 (13.3)	48 (15.6)	59 (24.3)	244 (15.4)	
Has at least one typical symptom	31 (3.0)	18 (5.8)	13 (5.3)	62 (3.9)	
**Having chronic diseases, n(%)**					0.526^a^
No	961 (93.1)	281 (91.2)	226 (93.0)	1468 (92.7)	
Yes	71 (6.9)	27 (8.8)	17 (7.0)	115 (7.3)	

Statistical comparison using

^a^ Chi-square test for categorical variable—display as n(%).

^b^ Fisher-exact test for categorical variable—display as n(%).

The **bold p-value** indicated statistical significance (p<0.05).

BMI: Body Mass Index, COVID-19: Coronavirus Disease of 2019.

The proportion screening positive for anxiety disorder among participants was 7.3%, (95% C.I.: 6.0–8.7), and the proportion of each severity level were 19.5% (95% C.I.:17.6–21.5) for mild group, 5.4% (95% C.I.: 4.4–6.6) for moderate group, and 1.9% (95% C.I.: 1.3–2.7) for severe group (**Table 2**). We observed differences in self-reported anxiety disorder among several variables: gender (p < 0.001), affordability of healthcare service (p < 0.001), and fear of COVID-19 (p < 0.001). In particular, the proportion of self-reported anxiety disorder among male students was higher than that among female students (10.5% vs. 5.3%, p < 0.001). The students who reported having difficulty paying for health services showed a higher level of self-reported anxiety disorder compared to the other group (4.7% vs. 10.0%, p < 0.001). Finally, the prevalence of self-reported anxiety disorder increased with the level of Fear of COVID-19 (Q4: 17.5% vs Q3: 9.0% vs Q2: 4.2% vs Q1: 3.7%, p < 0.001).

**Table 2:** The severity and screening for anxiety disorder.

	Self-reported anxiety disorder severity				Screening for anxiety disorder		P-value
	None	Mild	Moderate	Severe	Negative	Positive	
**Number of participants**	**1160**	**308**	**85**	**30**	**1468**	**115**	
**%** **(95% C.I.)**	**73.3** **(71.0–75.4)**	**19.5** **(17.6–21.5)**	**5.4** **(4.4–6.6)**	**1.9** **(1.3–2.7)**	**92.7** **(91.3–93.9)**	**7.3** **(6.0–8.7)**	
**Demographic factors**							
**Gender, n(%)**							**<0.001** ^**a**^
Female	739 (74.6)	199 (20.1)	39 (3.9)	14 (1.4)	938 (94.7)	53 (5.3)	
Male	421 (71.1)	109 (18.4)	46 (7.8)	16 (2.7)	530 (89.5)	62 (10.5)	
**Marital status, n(%)**							0.208^b^
Single	1152 (73.3)	306 (19.5)	85 (5.4)	28 (1.8)	1458 (92.8)	113 (7.2)	
Other	8 (66.7)	2 (16.7)	0 (0.0)	2 (16.7)	10 (83.3)	2 (16.7)	
**Having difficulty in paying for healthcare service, n(%)**							**<0.001** ^**a**^
No	662 (80.4)	122 (14.8)	32 (3.9)	7 (0.9)	784 (95.3)	39 (4.7)	
Yes	498 (65.5)	186 (24.5)	53 (7.0)	23 (3.0)	684 (90.0)	76 (10.0)	
**Academic factors**							
**Academic major, n(%)**							0.252^a^
Doctor of General Medicine	747 (72.4)	203 (19.7)	64 (6.2)	18 (1.7)	950 (92.1)	82 (7.9)	
Doctor of Preventive Medicine	228 (74.0)	59 (19.2)	15 (4.9)	6 (1.9)	287 (93.2)	21 (6.8)	
Bachelor of Nursing	185 (76.1)	46 (18.9)	6 (2.5)	6 (2.5)	231 (95.1)	12 (4.9)	
**Having clinical experience, n(%)**							0.394^a^
No	396 (72.3)	108 (19.7)	31 (5.7)	13 (2.4)	504 (92.0)	44 (8.0)	
Yes	764 (73.8)	200 (19.3)	54 (5.2)	17 (1.6)	964 (93.1)	71 (6.9)	
**Health factors, n(%)**							
**BMI classification, n(%)**							0.126^a^
Underweight	224 (69.6)	74 (23.0)	16 (5.0)	8 (2.5)	298 (92.5)	24 (7.5)	
Normal	760 (74.1)	195 (19.0)	52 (5.1)	19 (1.9)	955 (93.1)	71 (6.9)	
Overweight	157 (75.1)	37 (17.7)	12 (5.7)	3 (1.4)	194 (92.8)	15 (7.2)	
Obese	19 (73.1)	2 (7.7)	5 (19.2)	0 (0.0)	21 (80.8)	5 (19.2)	
**COVID-19 symptoms, n(%)**							0.355^a^
Has no symptoms	965 (75.6)	224 (17.5)	65 (5.1)	23 (1.8)	1189 (93.1)	88 (6.9)	
Has only atypical symptoms	157 (64.3)	67 (27.5)	18 (7.4)	2 (0.8)	224 (91.8)	20 (8.2)	
Has at least one typical symptom	38 (61.3)	17 (27.4)	2 (3.2)	5 (8.1)	55 (88.7)	7 (11.3)	
**Having chronic diseases, n(%)**							0.539^a^
No	1081 (73.6)	282 (19.2)	77 (5.2)	28 (1.9)	1363 (92.8)	105 (7.2)	
Yes	79 (68.7)	26 (22.6)	8 (7.0)	2 (1.7)	105 (91.3)	10 (8.7)	
**Fear of COVID-19, n(%)**							**<0.001** ^**a**^
Q1	350 (81.4)	64 (14.9)	11 (2.6)	5 (1.2)	414 (96.3)	16 (3.7)	
Q2	294 (77.2)	71 (18.6)	14 (3.7)	2 (0.5)	365 (95.8)	16 (4.2)	
Q3	420 (68.6)	137 (22.4)	44 (7.2)	11 (1.8)	557 (91.0)	55 (9.0)	
Q4	96 (60.0)	36 (22.5)	16 (10.0)	12 (7.5)	132 (82.5)	28 (17.5)	

Statistical comparison between screened negative and positive using.

^a^ Chi-square test for categorical variable—display as n(%).

^b^ Fisher-exact test for categorical variable—display as n(%).

The **bold p-value** indicated statistical significance (p<0.05).

BMI: Body Mass Index, COVID-19: Coronavirus Disease of 2019, the FCoV-19S score was divided into four groups based on quartiles: Q1, Q2, Q3, and Q4. Q1 group contained 25% participants with the lowest score from 7 to 13 points; Q2 group consisted of 25% participants with medium low score from 14 to 20 points; Q3 group was 25% participants with medium high score from 21 to 27 points; Q4 group was 25% participants with highest score from 28 to 35 points.

The prevalence of students screening positively for depression was 14.5%, (95% C.I.: 12.8–16.3), and the prevalence of each self-reported depression level was 28.6% (95% C.I.: 26.4–30.8) for mild, 8.3% (95% C.I.: 7.0–9.7) for moderate, 4.6% (95% C.I.: 3.7–5.8) for moderately severe, and 1.6% (95% C.I.: 1.1–2.3) for severe (**Table 3**). We also detected differences in the prevalence of self-reported depression among groups in relation to affordability of Health Service (p < 0.001), COVID-19 symptoms (p = 0.026), chronic diseases status (p = 0.043), and Fear of COVID-19 (p < 0.001). In particular, the proportion of self-reported depression among students having limited finance for health service was significantly higher than the proportion among the other group (19.2% vs. 10.1%, p < 0.001). Those having at least one atypical symptom of COVID-19 possessed the highest prevalence of self-reported depression compared to students having only atypical symptoms or having no symptoms (21.0% vs. 18.9% and 13.3%, p = 0.026). Additionally, the probability of being screened positive with depression was also higher among students with chronic diseases (20.9% vs. 14.0%, p = 0.043) and seemed to increase with the level of Fear of COVID-19 (Q4: 25.6% vs Q3: 16.2% vs Q2: 10.8% vs Q1: 11.2%, p < 0.001).

**Table 3:** The severity and screening for depression.

	Self-reported depression severity					Screening for depression		P-value
	None	Mild	Moderate	Moderately severe	Severe	Negative	Positive	
**Number of participants**	**902**	**452**	**131**	**73**	**25**	**1354**	**229**	
**% (95% C.I.)**	**57.0 (54.5–59.4)**	**28.6 (26.4–30.8)**	**8.3 (7.0–9.7)**	**4.6** **(3.7–5.8)**	**1.6 (1.1–2.3)**	**85.5** **(83.7–87.2)**	**14.5** **(12.8–16.3)**	
**Demographic factors**								
**Gender, n(%)**								0.167^a^
Female	553 (55.8)	304 (30.7)	82 (8.3)	40 (4.0)	12 (1.2)	857 (86.5)	134 (13.5)	
Male	349 (59.0)	148 (25.0)	49 (8.3)	33 (5.6)	13 (2.2)	497 (84.0)	95 (16.0)	
**Marital status, n(%)**								0.828^b^
Single	894 (56.9)	450 (28.6)	131 (8.3)	73 (4.6)	23 (1.5)	1344 (85.6)	227 (14.4)	
Other	8 (66.7)	2 (16.7)	0 (0.0)	0 (0.0)	2 (16.7)	10 (83.3)	2 (16.7)	
**Having difficulty in paying for healthcare service, n(%)**								**<0.001** ^**a**^
No	527 (64.0)	213 (25.9)	55 (6.7)	19 (2.3)	9 (1.1)	740 (89.9)	83 (10.1)	
Yes	375 (49.3)	239 (31.4)	76 (10.0)	54 (7.1)	16 (2.1)	614 (80.8)	146 (19.2)	
**Academic factors**								
**Academic major, n(%)**								0.489^a^
Doctor of General Medicine	584 (56.6)	291 (28.2)	90 (8.7)	51 (4.9)	16 (1.6)	875 (84.8)	157 (15.2)	
Doctor of Preventive Medicine	171 (55.5)	98 (31.8)	20 (6.5)	15 (4.9)	4 (1.3)	269 (87.3)	39 (12.7)	
Bachelor of Nursing	147 (60.5)	63 (25.9)	21 (8.6)	7 (2.9)	5 (2.1)	210 (86.4)	33 (13.6)	
**Having clinical experience, n(%)**								0.275^a^
No	316 (57.7)	160 (29.2)	38 (6.9)	24 (4.4)	10 (1.8)	476 (86.9)	72 (13.1)	
Yes	586 (56.6)	292 (28.2)	93 (9.0)	49 (4.7)	15 (1.4)	878 (84.8)	157 (15.2)	
**Health factors**								
**BMI classification, n(%)**								0.705^a^
Underweight	170 (52.8)	104 (32.3)	24 (7.5)	19 (5.9)	5 (1.6)	274 (85.1)	48 (14.9)	
Normal	586 (57.1)	298 (29.0)	85 (8.3)	41 (4.0)	16 (1.6)	884 (86.2)	142 (13.8)	
Overweight	130 (62.2)	45 (21.5)	21 (10.0)	9 (4.3)	4 (1.9)	175 (83.7)	34 (16.3)	
Obese	16 (61.5)	5 (19.2)	1 (3.8)	4 (15.4)	0 (0.0)	21 (80.8)	5 (19.2)	
**COVID-19 symptoms, n(%)**								**0.026** ^**a**^
Has no symptoms	769 (60.2)	338 (26.5)	95 (7.4)	53 (4.2)	22 (1.7)	1107 (86.7)	170 (13.3)	
Has only atypical symptoms	106 (43.4)	92 (37.7)	29 (11.9)	15 (6.1)	2 (0.8)	198 (81.1)	46 (18.9)	
Has at least one typical symptom	27 (43.5)	22 (35.5)	7 (11.3)	5 (8.1)	1 (1.6)	49 (79.0)	13 (21.0)	
**Having chronic diseases, n(%)**								**0.043** ^**a**^
No	852 (58.0)	411 (28.0)	116 (7.9)	66 (4.5)	23 (1.6)	1263 (86.0)	205 (14.0)	
Yes	50 (43.5)	41 (35.7)	15 (13.0)	7 (6.1)	2 (1.7)	91 (79.1)	24 (20.9)	
**Fear of COVID-19, n(%)**								**<0.001** ^**a**^
Q1	269 (62.6)	113 (26.3)	34 (7.9)	9 (2.1)	5 (1.2)	382 (88.8)	48 (11.2)	
Q2	221 (58.0)	119 (31.2)	26 (6.8)	13 (3.4)	2 (0.5)	340 (89.2)	41 (10.8)	
Q3	333 (54.4)	180 (29.4)	55 (9.0)	37 (6.0)	7 (1.1)	513 (83.8)	99 (16.2)	
Q4	79 (49.4)	40 (25.0)	16 (10.0)	14 (8.8)	11 (6.9)	119 (74.4)	41 (25.6)	

Statistical comparison between screened negative and positive using.

^a^ Chi-square test for categorical variable—display as n(%).

^b^ Fisher-exact test for categorical variable—display as n(%).

The **bold p-value** indicated statistical significance (p<0.05).

BMI: Body Mass Index, COVID-19: Coronavirus Diseases of 2019, the FCoV-19S score was divided into four groups based on quartiles: Q1, Q2, Q3, and Q4. Q1 group contained 25% participants with the lowest score from 7 to 13 points; Q2 group consisted of 25% participants with medium low score from 14 to 20 points; Q3 group was 25% participants with medium high score from 21 to 27 points; Q4 group was 25% participants with highest score from 28 to 35 points.

The prevalence of students perceiving worsening mental health during COVID-19 was 6.9% (95% C.I.: 5.7–8.3) (**Table 4**). Factors that showed the significant association included clinical experience (p = 0.007), COVID-19 symptoms (p = 0.001), and chronic disease (p = 0.002). Specifically, students with clinical experience were more likely to perceive worsening mental health than those with no clinical experience did (8.2% vs. 4.6%, p = 0.007). Among COVID-19 symptom groups, those with atypical COVID-19 symptoms or at least one typical COVID-19 symptom were more likely to perceive worsening mental health than students with no symptoms (12.3% vs. 9.7% vs. 5.8%, p = 0.001). In addition, students having chronic disease presented a higher prevalence of perceived worsening mental health status than the other group (13.9% vs. 6.9%, p = 0.002).

**Table 4:** The distribution of students having worsening mental health.

Perception of worsening mental health	Not worsening	Worsening	P-value
**Number of participants**	**1473**	**110**	
**% (95% C.I.)**	**93.1 (91.7–94.2)**	**6.9 (5.7–8.3)**	
**Demographic factors**			
**Gender, n(%)**			0.860^a^
Female	923 (93.1)	68 (6.9)	
Male	550 (92.9)	42 (7.1)	
**Marital status, n(%)**			0.850^b^
Single	1462 (93.1)	109 (6.9)	
Other	11 (91.7)	1 (8.3)	
**Having difficulty in paying for healthcare service, n(%)**			0.305^a^
No	771 (93.7)	52 (6.3)	
Yes	702 (92.4)	58 (7.6)	
**Academic factors**			
**Academic major, n(%)**			
Doctor of General Medicine	964 (93.4)	68 (6.6)	0.651^a^
Doctor of Preventive Medicine	283 (91.9)	25 (8.1)	
Bachelor of Nursing	226 (93.0)	17 (7.0)	
**Having clinical experience, n(%)**			**0.007** ^**a**^
No	523 (95.4)	25 (4.6)	
Yes	950 (91.8)	85 (8.2)	
**Health factors**			
**BMI classification, n(%)**			0.810^a^
Underweight	301 (93.5)	21 (6.5)	
Normal	954 (93.0)	72 (7.0)	
Overweight	195 (93.3)	14 (6.7)	
Obese	23 (88.5)	3 (11.5)	
**COVID-19 symptoms, n(%)**			**0.001** ^**a**^
Has no symptoms	1203 (94.2)	74 (5.8)	
Has only atypical symptoms	214 (87.7)	30 (12.3)	
Has at least one typical symptom	56 (90.3)	6 (9.7)	
**Having chronic diseases, n(%)**			**0.002** ^**a**^
No	1374 (93.6)	94 (6.4)	
Yes	99 (86.1)	16 (13.9)	
**Fear of COVID-19, n(%)**			0.207^a^
Q1	407 (94.7)	23 (5.3)	
Q2	349 (91.6)	32 (8.4)	
Q3	572 (93.5)	40 (6.5)	
Q4	145 (90.6)	15 (9.4)	

Statistical comparison using.

^a^ Chi-square test for categorical variable—display as n(%).

^b^ Fisher-exact test for categorical variable—display as n(%).

The **bold p-value** indicated statistical significance (p<0.05).

BMI: Body Mass Index, COVID-19: Coronavirus Disease of 2019, the FCoV-19S score was divided into four groups based on quartiles: Q1, Q2, Q3, and Q4. Q1 group contained 25% participants with the lowest score from 7 to 13 points; Q2 group consisted of 25% participants with medium low score from 14 to 20 points; Q3 group was 25% participants with medium high score from 21 to 27 points; Q4 group was 25% participants with highest score from 28 to 35 points.

**Fig 1:**
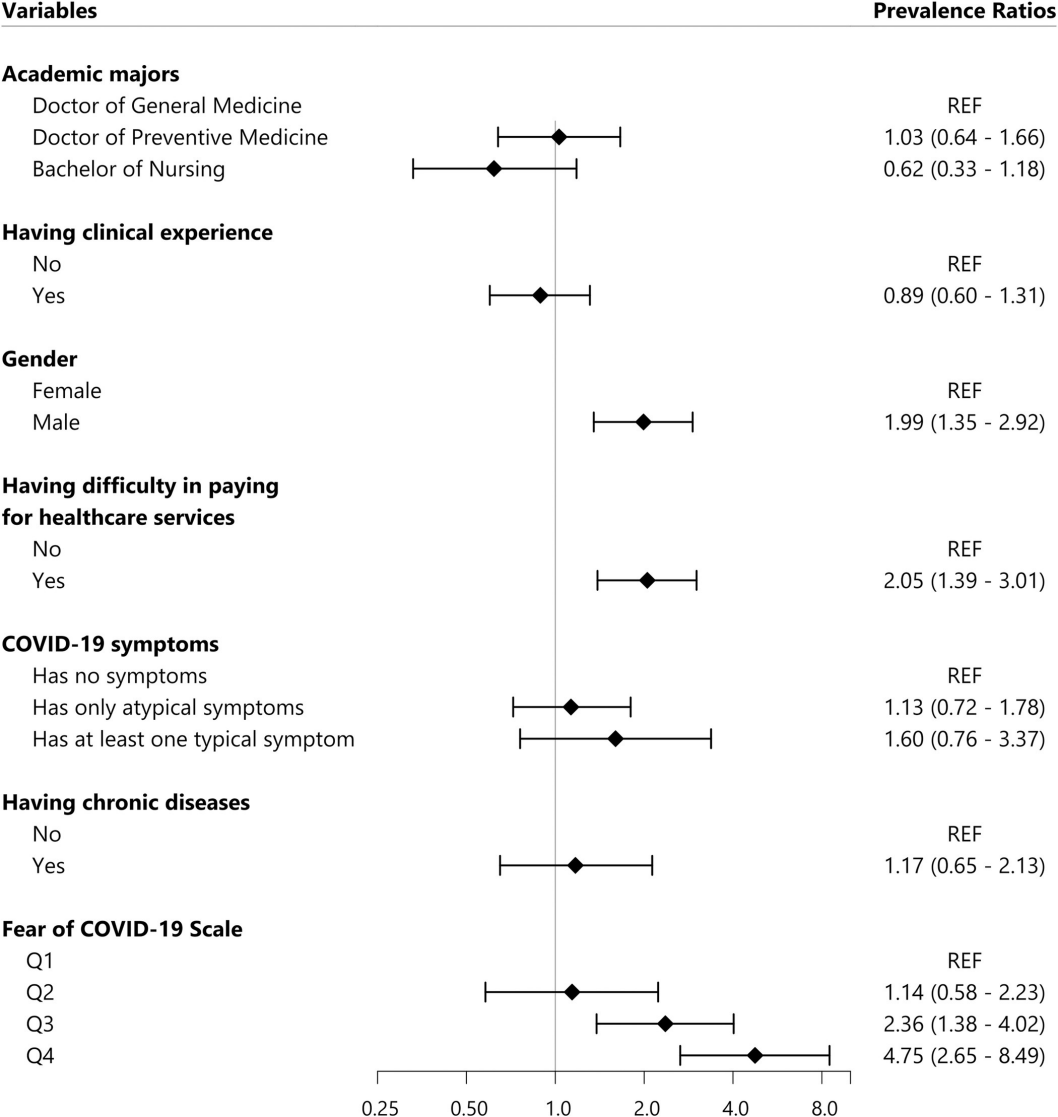
Factors associated with self-reported anxiety disorder. All Prevalence Ratios were adjusted using Poisson multivariate regression model. REF: Reference value, COVID-19: Coronavirus Diseases of 2019, the FCoV-19S score was divided into four groups based on quartiles: Q1, Q2, Q3, and Q4. Q1 group contained 25% participants with the lowest score from 7 to 13 points; Q2 group consisted of 25% participants with medium low score from 14 to 20 points; Q3 group was 25% participants with medium high score from 21 to 27 points; Q4 group was 25% participants with highest score from 28 to 35 points.

In anxiety disorder multivariable regression model (**Fig 1**), factors significantly associated with the prevalence of self-reported anxiety disorder included being male (PR = 1.99, 95% C.I.: 1.35–2.92), having difficultly affording for health care service (PR = 2.05, 95% C.I.: 1.39–3.01), and having with high level of Fear of COVID-19 (Q3: PR = 2.36, 95% C.I.: 1.38–4.02 and Q4: PR = 4.75, 95% C.I.: 2.65–8.49).

**Fig 2:**
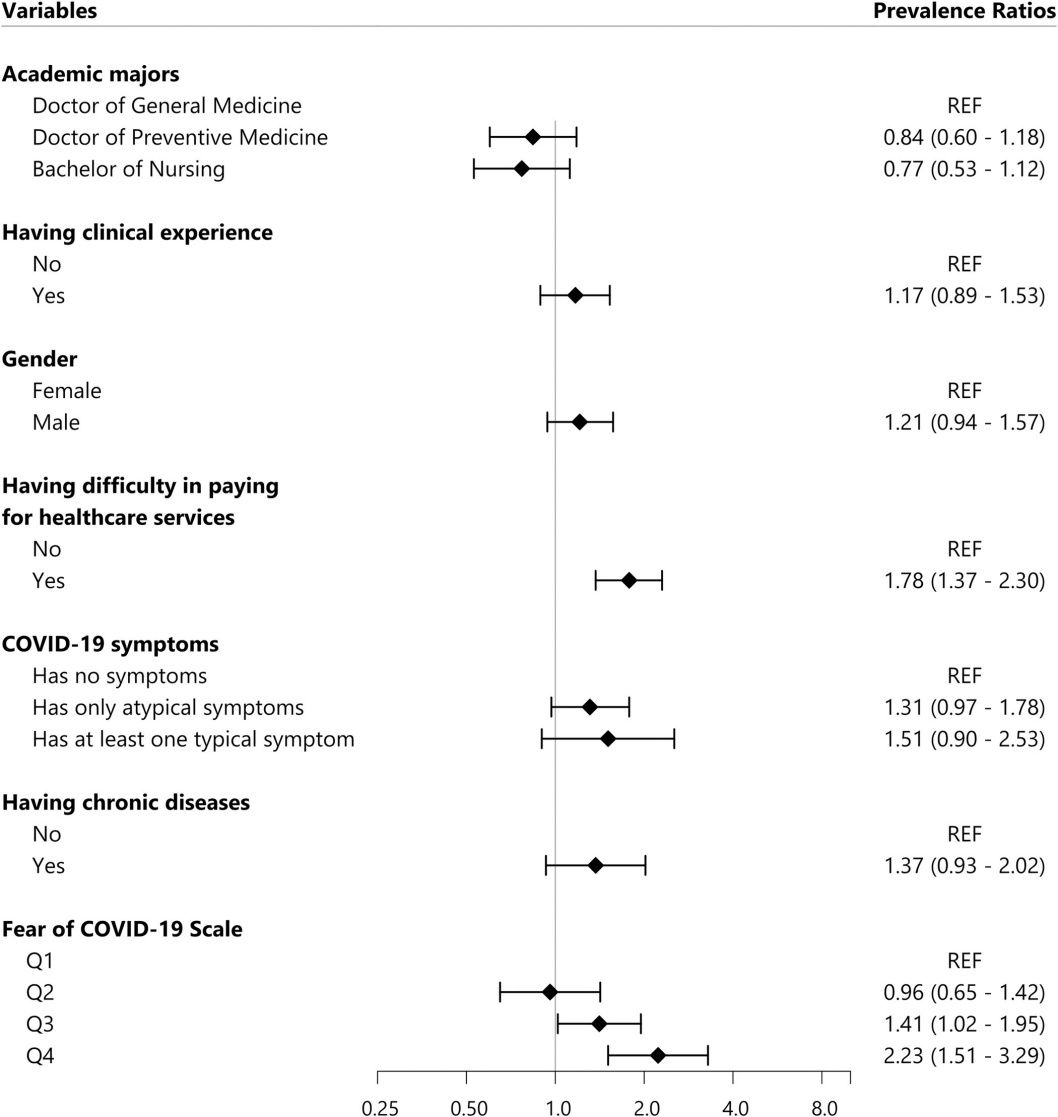
Factors associated with self-reported depression. All Prevalence Ratios were adjusted using Poisson multivariate regression model. REF: Reference value, COVID-19: Coronavirus Diseases of 2019, the FCoV-19S score was divided into four groups based on quartiles: Q1, Q2, Q3, and Q4. Q1 group contained 25% participants with the lowest score from 7 to 13 points; Q2 group consisted of 25% participants with medium low score from 14 to 20 points; Q3 group was 25% participants with medium high score from 21 to 27 points; Q4 group was 25% participants with highest score from 28 to 35 points.

Regarding the depression multivariable regression model (**Fig 2**), we found that students with difficult affording health service were more likely to be screened positive with depression (PR = 1.78, 95% C.I.: 1.37–2.30). In addition, the level of Fear of COVID-19 was also significantly associated with self-reported depression (Q3: PR = 1.41, 95% C.I.: 1.02–1.95 and Q4: PR = 2.23, 95% C.I.: 1.51–3.29).

**Fig 3:**
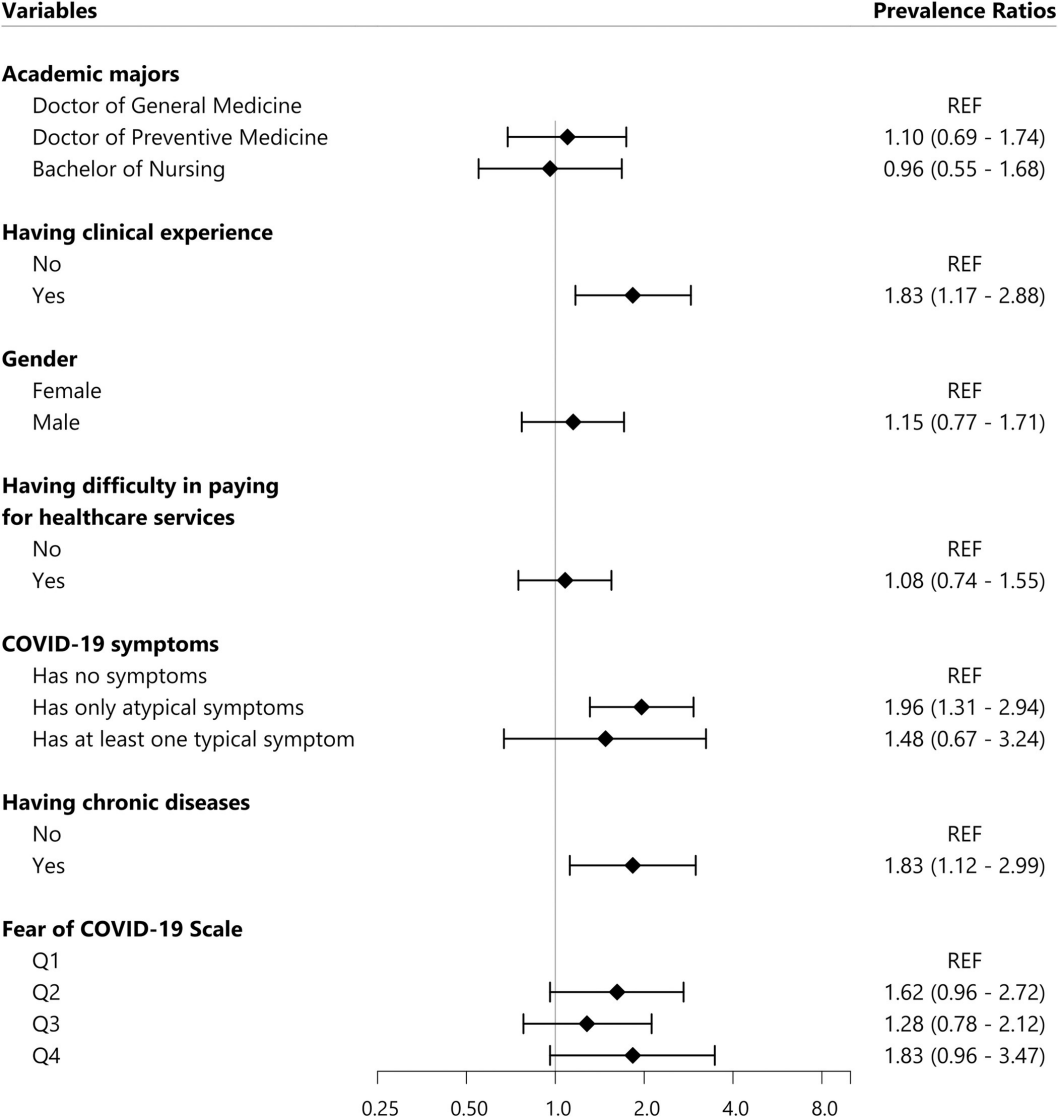
Factors associated with the worsening mental health status. All Prevalence Ratios were adjusted using Poisson multivariate regression model. REF: Reference value, COVID-19: Coronavirus Diseases of 2019, the FCoV-19S score was divided into four groups based on quartiles: Q1, Q2, Q3, and Q4. Q1 group contained 25% participants with the lowest score from 7 to 13 points; Q2 group consisted of 25% participants with medium low score from 14 to 20 points; Q3 group was 25% participants with medium high score from 21 to 27 points; Q4 group was 25% participants with highest score from 28 to 35 points.

Factors significantly associated with the worsening mental health status during COVID-19 pandemic included having clinical experience (PR = 1.83, 95% C.I.: 1.17–2.88) and having atypical symptoms of COVID-19 (PR = 1.96, 95% C.I.: 1.31–2.94) (**Fig 3**).

For sensitivity analysis, we conducted the same regression models on each group of academic majors (**S1–S3 Tables**) and compared the robust Poisson models to the logistic regression model (**S4-S5-S6 Tables in S1 File**).

## Discussion

Our study shows that the prevalence of students screened positive for depression was 14.5% (95% C.I.: 12.8–16.3), for anxiety disorder was 7.3% (95% C.I.: 6.0–8.7), and for perceiving their mental health to be worse due to the pandemic was 6.9% (95% C.I.: 5.7–8.3).

The prevalence of self-reported anxiety (7.3%; 95% C.I.: 6.0–8.7) in our survey was lower than the prevalence among students in Bezmialem Vakif, Turkey in June 2020 (37.1%) that was also utilized GAD-7 to screen [32]. A possible explanation is that the number of COVID-19 cases in Turkey in June 2020 was more than 160,000, much more than in Vietnam at the time of our study (in April 2020) with 270 total confirmed cases on April 30th, 2020 [32,33]. In addition, a study conducted by La Ngoc Quang and colleagues on Vietnamese healthcare workers in Dong Da General Hospital and Dong Anh General Hospital in Hanoi showed that the prevalence of moderate and severe anxiety disorder (GAD-7 score > 10) was 2.9%, which was lower than this prevalence in our research [24]. Our study results also presented that male students were 1.45 times (PR = 1.45, 95% C.I.: 1.17–1.80) more likely to be screened positive for anxiety disorder than female students, which was inconsistent with the literature [34–36]. This result could be explained by the fact that male students faced heavier workload due to COVID-19 outbreak as, according to our observation in Vietnam, they were prioritized to be recruited for COVID-19 response or performed hard and dangerous tasks.

The prevalence of self-reported depression (14.5%; 95% C.I.: 12.8–16.3) among Hanoi Medical University’s students in this study was similar to pre-pandemic level (15.2%) [15]. However, the result from a meta-analysis conducted by Tianchen Wu and colleagues showed that 32.9% (95% C.I.: 26.9–39.2) of front-line and second-line medical workers were screened positive with depression [37]. The higher proportion of depression reported by Tianchen Wu and colleagues could be explained that COVID-19 pandemic in Vietnam at the time of our study (from April 7^th^ to 29^th^, 2020) was not as severe as in other areas of the world (280 cases and zero death in Vietnam of total 3,090,445 cases and 217,769 deaths in the world on April 30^th^, 2020) [33]. Moreover, our sample was small and limited to one university in Hanoi, so there could be variation when comparing to larger studies and meta-analysis.

Additionally, 6.9% (95% C.I.: 5.7–8.3) of participants in our research believed that their mental health had been negatively affected by the pandemic outbreak. This result was consistent with a rapid review and meta-analysis conducted to examine the psychological effects on healthcare workers during such pandemics as Middle East Respiratory syndrome, Ebola virus diseases, COVID-19, H1N1, H7N9 [38]. The review presented that those in close contact with affected patients were more likely to suffer from psychological distress (OR = 1.74, 95% C.I.: 1.50–2.03) [38]. Moreover, this impact appeared during and after the pandemic outbreak, persisting up to three years later [38,39]. Perceiving worsening mental health status during the pandemic was significantly associated with clinical experience (PR = 1.83, 95% C.I.: 1.17–2.88) and COVID-19 atypical symptoms (PR = 1.96, 95% C.I.: 1.31–2.94). This result could be explained by the fact that students might be under more pressure during clinical rotation in the hospital or have symptoms of COVID-19 during the pandemic. The pressure also came from the fear of being exposed with novel coronavirus or the overcrowding in hospital when the number of COVID-19 cases kept increasing [10]. Consequently, providing support to affected medical students during their clinical rotation in the pandemic may relieve some of these mental health issues. Moreover, the universities and hospitals could provide students more training regarding COVID-19 prevention measure, so all students would feel safer when working in hospital.

Fear of COVID-19 also showed the significant association with self-reported depression and anxiety disorder. Students with highest score of Fear of COVID-19 Scale (the Q4 group) would be 4.75 times (PR = 4.75, 95% C.I.: 2.65–8.49) more likely to be screened positive with anxiety disorder and 2.23 times (PR = 2.23, 95% C.I.: 1.51–3.29) more likely to be screened positive with depression compared to those having the lowest Fear of COVID-19 Scale score (the Q1 group). This was not a surprise finding because COVID-19 had also impacted negatively on the mental health of medical students and the global population as well [11,40,41]. Consequently, fear and anxiety about the pandemic, such as worrying about acquiring SARS-CoV-2, dying of COVID-19, or negative news about the pandemic, may lead to an increase in the prevalence of anxiety disorder and depression [42–44].

Finally, we observed the associations between the affordability of healthcare service and both self-reported depression (PR = 1.78, 95% C.I.: 1.37–2.30) and anxiety disorder (PR = 2.05, 95% C.I.: 1.39–3.01). This finding was consistent with previous results in Vietnam [15], which showed participants with financial burdens were nearly twice as likely to be screened positive for depression [15]. During the pandemic, financial burden may create even more negative impact on medical students’ mental health due to increased healthcare cost associated with COVID-19 testing, treatment and other costs, such as lodging, during the mobilization effort to control the pandemic. As a result, the government and universities may need consider providing financial support for students, especially those were mobilized to help with the COVID-19 response [45].

The strength of our study was that all students in Doctor of General Medicine, Doctors of Preventive Medicine, and Bachelor of Nursing programs were invited participate in the survey. Additionally, this study used PHQ-9 and GAD-7 to screen for depression and anxiety disorder with high sensitivity (88% for PHQ-9 and 89% for GAD-7) and specificity (88% for PHQ-9 and 82% for GAD-7). We also conducted this study in April 2020, immediately after Bach Mai hospital, one of the vital teaching hospitals of Hanoi Medical University, was locked down due to a COVID-19 case series on March 28^th^, 2020. The study captures the mental health status of Hanoi Medical University’s students at a critical time during the pandemic. However, the limitation of the research mostly lies on the low response rate (in this study: 43.1%) that possibly caused sampling errors and selection bias. In addition, the mental health screening questionnaires for depression and anxiety disorder detect symptoms of those conditions rather than definitive clinical diagnoses. Similarly, we utilized a single direct question to examine worsening mental health status, so it was difficult to define accurately the change in mental health status among students. The translation of FcoV-19S, as well as other English questionnaires, may decrease their validity. Besides, information related to students’ health, such as height, weight, and chronic disease was self-reported, and many important information, such as mental health history, workload, and study load were not collected. Finally, the cross-sectional study could only detect factors associated with mental health variables rather than present clear causal relationships. This design also prevented us from looking at mental health status during other waves of COVID-19 in Vietnam.

## Conclusion

In conclusion, this survey shows that the prevalence of self-reported depression (14.5%), anxiety disorder (7.3%), and worsening mental health (6.9%) among Hanoi Medical University’s students was lower than in medical students in other areas in the world. The prevalence of self-reported depression was similar to pre-pandemic level in Vietnam. Key factors associated with mental health status of medical students during COVID-19 pandemic included the affordability of healthcare services, clinical experience, and the fear of COVID-19. We recommend that, medical universities consider feasible solutions to improve the students’ mental health status during the outbreak of pandemics such as providing basic healthcare service for students in need, supporting students with healthcare service cost, adjusting clinical study program to reduce stress and exposure to COVID-19, or providing students with overall knowledge about the prevention for COVID-19 in clinical settings.

## Supporting information

S1 Table Regression models of self-reported anxiety disorder on each group of academic majors. (DOCX)

S2 Table Regression models of self-reported depression on each group of academic majors. (DOCX)

S3 Table Regression models of worsening mental health on each group of academic majors. (DOCX)

S1 File Comparison of Logistic regression and Poisson regression of self-reported anxiety disorder, self-reported depression, and worsening mental health. (XLSX)
