# Supplementary material for: The prevalence of self-reported anxiety, depression, and associated factors among Hanoi Medical University’s students during the first wave of COVID-19 pandemic
Source: PLoS One. 2022 Aug 12;17(8):e0269740. doi: 10.1371/journal.pone.0269740 (PMC9374228; doi:10.1371/journal.pone.0269740)
Supplement: S1 Table — (DOCX) [file pone.0269740.s001.docx]

|  | **Prevalence Ratios (95% C.I.) of Anxiety Disorder** | | | |
| --- | --- | --- | --- | --- |
| **Variables** | **Total Sample** | **Doctor of General Medicine** | **Doctor of Preventive Medicine** | **Nurse** |
| **Academic majors** |  |  |  |  |
| Doctor of General Medicine | REF | N/A | N/A | N/A |
| Doctor of Preventive Medicine | 1.03 (0.64 ─ 1.66) | N/A | N/A | N/A |
| Nurse | 0.62 (0.33 ─ 1.18) | N/A | N/A | N/A |
| **Having clinical experience** |  |  |  |  |
| No | REF | REF | REF | REF |
| Yes | 0.89 (0.60 ─ 1.31) | 0.91 (0.58 ─ 1.42) | 0.38 (0.17 ─ 0.86) | 3.28 (0.69 ─ 15.5) |
| **Gender** |  |  |  |  |
| Female | REF | REF | REF | REF |
| Male | **1.99 (1.35 ─ 2.92)** | **1.66 (1.09 ─ 2.53)** | **3.94 (1.76 ─ 8.83)** | 3.02 (0.68 ─ 13.3) |
| **Having difficulty in paying for healthcare services** |  |  |  |  |
| No | REF | REF | REF | REF |
| Yes | **2.05 (1.39 ─ 3.01)** | **1.68 (1.08 ─ 2.62)** | **4.24 (1.58 ─ 11.40)** | 2.95 (0.64 ─ 13.6) |
| **COVID-19 symptoms** |  |  |  |  |
| Has no symptoms | REF | REF |  | REF |
| Has only atypical symptoms | 1.13 (0.72 ─ 1.78) | 1.00 (0.54 – 1.84) | 1.11 (0.46 ─ 2.66) | 1.36 (0.40 ─ 4.61) |
| Has at least one typical symptom | 1.60 (0.76 ─ 3.37) | **2.40 (1.10 – 5.22)** | 1.00 (0.13 ─ 7.64) | N/A |
| **Having chronic diseases** |  |  |  |  |
| No | REF | REF | REF | REF |
| Yes | 1.17 (0.65 ─ 2.13) | 0.79 (0.33 ─ 1.90) | **2.85 (1.13 ─ 7.19)** | 1.4 (0.21 ─ 9.32) |
| **Fear of COVID-19 Scale** |  |  |  |  |
| Q1 | REF | REF | REF | REF |
| Q2 | 1.14 (0.58 ─ 2.23) | 1.04 (0.49 ─ 2.24) | 1.36 (0.25 ─ 7.31) | 0.50 (0.03 ─ 7.80) |
| Q3 | **2.36 (1.38 ─ 4.02)** | **2.14 (1.16 ─ 3.94)** | 2.54 (0.68 ─ 9.49) | 1.28 (0.17 ─ 9.55) |
| Q4 | **4.75 (2.65 ─ 8.49)** | **4.77 (2.46 ─ 9.23)** | **4.76 (1.20 ─ 18.90)** | 2.25 (0.27 ─ 18.7) |
|  | | | | |
| REF: reference value | | | | |
| N/A: not applicable | | | | |
| The bold Prevalence Ratio and 95% C.I. presents the statistical significance | | | | |

**S1 Table. Regression models of Anxiety Disorder on each group of Academic majors**
