# Supplementary material for: The prevalence of self-reported anxiety, depression, and associated factors among Hanoi Medical University’s students during the first wave of COVID-19 pandemic
Source: PLoS One. 2022 Aug 12;17(8):e0269740. doi: 10.1371/journal.pone.0269740 (PMC9374228; doi:10.1371/journal.pone.0269740)
Supplement: S2 Table — (DOCX) [file pone.0269740.s002.docx]

**S2 Table. Regression models of Depression on each group of Academic majors**

|  | **Prevalence Ratios (95% C.I.) of Depression** | | | |
| --- | --- | --- | --- | --- |
| **Variables** | **Total Sample** | **Doctor of General Medicine** | **Doctor of Preventive Medicine** | **Nurse** |
| **Academic majors** |  |  |  |  |
| Doctor of General Medicine | REF | N/A | N/A | N/A |
| Doctor of Preventive Medicine | 0.84 (0.601 ─ 1.18) | N/A | N/A | N/A |
| Nurse | 0.77 (0.531 ─ 1.12) | N/A | N/A | N/A |
| **Having clinical experience** |  |  |  |  |
| No | REF | REF | REF | REF |
| Yes | 1.17 (0.892 ─ 1.53) | 1.19 (0.864 ─ 1.64) | 0.892 (0.465 ─ 1.71) | 1.33 (0.713 ─ 2.49) |
| **Gender** |  |  |  |  |
| Female | REF |  |  |  |
| Male | 1.21 (0.935 ─ 1.57) | 1.09 (0.824 ─ 1.45) | **1.99 (1.11 ─ 3.57)** | 1.22 (0.235 ─ 6.35) |
| **Having difficulty in paying for healthcare services** |  |  |  |  |
| No | REF |  |  |  |
| Yes | **1.78 (1.37 ─ 2.3)** | **1.78 (1.29 ─ 2.44)** | 1.71 (0.926 ─ 3.16) | 1.68 (0.825 ─ 3.43) |
| **COVID-19 symptoms** |  |  |  |  |
| Has no symptoms | REF |  |  |  |
| Has only atypical symptoms | 1.31 (0.969 ─ 1.78) | 1.13 (0.754 ─ 1.7) | 1.44 (0.682 ─ 3.06) | 1.76 (0.881 ─ 3.51) |
| Has at least one typical symptom | 1.51 (0.9 ─ 2.53) | 1.79 (0.976 ─ 3.29) | 0.517 (0.0704 ─ 3.79) | 2.2 (0.672 ─ 7.21) |
| **Having chronic diseases** |  |  |  |  |
| No | REF |  |  |  |
| Yes | 1.37 (0.931 ─ 2.02) | 1.29 (0.783 ─ 2.11) | 1.61 (0.715 ─ 3.63) | 1.23 (0.41 ─ 3.66) |
| **Fear of COVID-19 Scale** |  |  |  |  |
| Q1 | REF |  |  |  |
| Q2 | 0.96 (0.65 ─ 1.42) | 0.963 (0.618 ─ 1.5) | 0.728 (0.216 ─ 2.45) | 1.27 (0.303 ─ 5.31) |
| Q3 | **1.41 (1.02 ─ 1.95)** | 1.27 (0.868 ─ 1.86) | 2 (0.896 ─ 4.48) | 1.51 (0.426 ─ 5.33) |
| Q4 | **2.23 (1.51 ─ 3.29)** | **2.11 (1.32 ─ 3.36)** | **2.81 (1.06 ─ 7.46)** | 2.37 (0.66 ─ 8.51) |
|  | | | | |
| REF: reference value | | | | |
| N/A: not applicable | | | | |
| The bold Prevalence Ratio and 95% C.I. presents the statistical significance | | | | |
