# Supplementary material for: The prevalence of self-reported anxiety, depression, and associated factors among Hanoi Medical University’s students during the first wave of COVID-19 pandemic
Source: PLoS One. 2022 Aug 12;17(8):e0269740. doi: 10.1371/journal.pone.0269740 (PMC9374228; doi:10.1371/journal.pone.0269740)
Supplement: S3 Table — (DOCX) [file pone.0269740.s003.docx]

**S3 Table. Regression models of worsen mental health on each group of Academic majors**

|  | **Prevalence Ratios (95% C.I.) of perceiving of worsen mental health** | | | |
| --- | --- | --- | --- | --- |
| **Variables** | **Total Sample** | **Doctor of General Medicine** | **Doctor of Preventive Medicine** | **Nurse** |
| **Academic majors** |  |  |  |  |
| Doctor of General Medicine | REF | N/A | N/A | N/A |
| Doctor of Preventive Medicine | 1.1 (0.694 ─ 1.74) | N/A | N/A | N/A |
| Nurse | 0.961 (0.55 ─ 1.68) | N/A | N/A | N/A |
| **Having clinical experience** |  |  |  |  |
| No | REF | REF | REF | REF |
| Yes | 1.83 (1.17 ─ 2.88) | 2.95 (1.58 ─ 5.49) | 1.86 (0.557 ─ 6.18) | 0.425 (0.167 ─ 1.08) |
| **Gender** |  |  |  |  |
| Female | REF | REF | REF | REF |
| Male | 1.15 (0.774 ─ 1.71) | 1.07 (0.683 ─ 1.68) | 1.7 (0.77 ─ 3.73) | N/A |
| **Having difficulty in paying for healthcare services** |  |  |  |  |
| No | REF | REF | REF | REF |
| Yes | 1.08 (0.747 ─ 1.55) | 1.11 (0.691 ─ 1.79) | 0.756 (0.367 ─ 1.56) | 1.27 (0.496 ─ 3.24) |
| **COVID-19 symptoms** |  |  |  |  |
| Has no symptoms | REF | REF | REF | REF |
| Has only atypical symptoms | 1.96 (1.31 ─ 2.94) | 1.22 (0.656 ─ 2.26) | 4.22 (1.83 ─ 9.73) | 2.49 (1 ─ 6.18) |
| Has at least one typical symptom | 1.48 (0.673 ─ 3.24) | 1.89 (0.735 ─ 4.84) | 1.8 (0.414 ─ 7.85) | N/A |
| **Having chronic diseases** |  |  |  |  |
| No | REF | REF | REF | REF |
| Yes | 1.83 (1.12 ─ 2.99) | 2.2 (1.18 ─ 4.13) | 1.58 (0.586 ─ 4.27) | 0.71 (0.11 ─ 4.58) |
| **Fear of COVID-19 Scale** |  |  |  |  |
| Q1 | REF | REF | REF | REF |
| Q2 | 1.62 (0.965 ─ 2.72) | 1.81 (0.978 ─ 3.34) | 1.62 (0.544 ─ 4.84) | 1.15 (0.204 ─ 6.46) |
| Q3 | 1.28 (0.777 ─ 2.12) | 1.13 (0.591 ─ 2.15) | 1.66 (0.575 ─ 4.79) | 1.14 (0.254 ─ 5.08) |
| Q4 | 1.83 (0.961 ─ 3.47) | 2.19 (0.981 ─ 4.9) | 2.32 (0.663 ─ 8.14) | 0.85 (0.15 ─ 4.69) |
|  | | | | |
| REF: reference value | | | | |
| N/A: not applicable | | | | |
| The bold Prevalence Ratio and 95% C.I. presents the statistical significance | | | | |
